# Computational Perspectives into Plasmepsins Structure—Function Relationship: Implications to Inhibitors Design

**DOI:** 10.1155/2011/657483

**Published:** 2011-07-03

**Authors:** Alejandro Gil L., Pedro A. Valiente, Pedro G. Pascutti, Tirso Pons

**Affiliations:** ^1^Laboratorio de Biología Computacional y Diseño de Proteínas, Centro de Estudio de Proteínas (CEP), Facultad de Biología, Universidad de La Habana, Cuba; ^2^Instituto de Biofísica Carlos Chagas Filho, Universidad Federal do Rio de Janeiro, Brazil

## Abstract

The development of efficient and selective antimalariais remains a challenge for the pharmaceutical industry. The aspartic proteases plasmepsins, whose inhibition leads to parasite death, are classified as targets for the design of potent drugs. Combinatorial synthesis is currently being used to generate inhibitor libraries for these enzymes, and together with computational methodologies have been demonstrated capable for the selection of lead compounds. The high structural flexibility of plasmepsins, revealed by their X-ray structures and molecular dynamics simulations, made even more complicated the prediction of putative binding modes, and therefore, the use of common computational tools, like docking and free-energy calculations. In this review, we revised the computational strategies utilized so far, for the structure-function relationship studies concerning the plasmepsin family, with special focus on the recent advances in the improvement of the linear interaction estimation (LIE) method, which is one of the most successful methodologies in the evaluation of plasmepsin-inhibitor binding affinity.

## 1. Introduction

More than 40% of the world's population lives with some risk of contracting malaria, with most recent estimates suggesting several hundred millions of clinical cases with 800,000 deaths each year [1]. In humans, the disease is the result of the infection by *Plasmodium falciparum *(Pf), *Plasmodium malariae*, *Plasmodium ovalae*, or *Plasmodium vivax*. Of these species, Pf is the most lethalm and it is, therefore, the main target for drug intervention. Once the microbe is transmitted to humans by mosquitoes of the anopheles genus, it causes many problems, but most commonly severe, recurring fever attacks [2]. Despite considerable efforts in this field, it has not been possible to develop an efficient vaccine to prevent malaria. The main disadvantages are (i) the increasing resistance of vectors to insecticides and (ii) the emergence of multidrug-resistant variants of Pf to existing antimalarial drugs, with the exception of the artemisinns [3]. Therefore, in the last years, researchers have focused their efforts towards the discovery of more selective and potent drugs [2].

Hemoglobin (Hb)-degrading enzymes of Pf emerge as very promising chemotherapeutic targets, because Hb degradation is a unique and critical process for Pf [2]. During the intraerythrocytic stage of the parasite's life cycle, this protozoon consumes approximately 75% of the Hb in the infected red blood cell [7, 8], which provides the main source of amino acids for the parasite growth and maturation [2]. The Hb degradation occurs within the acidic food vacuole (DV) of the parasite, and it is catalyzed by four aspartic proteases (plasmepsins) [9–11], three cysteine proteases (falcipains) [12–15], one metalloprotease (falcilicin) [16], and one dipeptidyl aminopeptidase 1 (DAPP1) [17]. Plasmepsin II (PlmII) has been the most extensively characterized of these enzymes, since several crystal structures have been determined [18–20] and potent inhibitors developed [21–24] (see [25] for a comprehensive review). However, most of these compounds have generally shown limited selectivity towards the human-related protease cathepsin D (hCatD) [25]. This feature is an important issue to point out to reduce the toxicity side effects when developing inhibitors of pathogenic enzymes [26]. On the other hand, the high degree of structural flexibility of the Plms active site cavity allows the accommodation of different inhibitors scaffolds [6]. This is a notable drawback for drug design using the traditional rigid docking approaches [27], due to the great challenge that constitutes the correct prediction of the inhibitor-binding mode and association free-energy.

In this review, we focus on the computational perspectives for plasmepsins drug design based on the sequence-structure-function relationship of these proteins, a major challenge in this field. To get a better understanding, we have divided the manuscript into four sections related with key steps of the traditional scheme, followed by virtual screening or drug design processes. In Section 1, we provide a brief description about the evolution of Plms as chemotherapeutic targets. In Section 2, we show the main findings provided by sequence and structural analysis of Plm family. In Section 3, we describe the most popular approaches used in the plasmepsin inhibitor design [27]. In Section 4, we provide a detailed description about the use of the linear interaction energy (LIE) method [30] in the refining steps of Plms inhibitor design.

## 2. Evolution of Plasmepsins as Chemotherapeutic Targets

The structure-based drug design of antimalarial compounds targeting plasmepsins inhibition has received much attention due to their potential biomedical use. Earlier studies indicated that Plms play essential roles in Pf life cycle, due to the effectiveness of Plms inhibitors abolishing Hb degradation, erythrocyte rupture, and parasite development. Indeed, pepstatin A, a nonspecific broad-range aspartic peptidase inhibitor, causes the death of the Plasmodium microbes when added to culture red cells infected with parasites [31, 32]. A similar behavior has been reported in animal models infected with Plasmodium parasite when E-64, a nonspecific broad-range cysteine peptidase inhibitor was administered, and both inhibitors displayed a synergic effect when combined [33–36]. 

Sequencing of the Pf genome has identified 10 Plms encoding genes, numbered PlmI to PlmX [26, 37]. Among these, only PlmI, PlmII, HAP (histoaspartic protease, or PlmIII), and PlmIV are active in the DV [38]. Although these four enzymes are capable of cleaving native and acid-denature hemoglobin at *α* chain L33-F34 site [8, 9, 38–40], PlmIV and HAP appears to prefer denature globin over the native protein [38]. PlmV, PlmIX and PlmX are expressed concurrently with PlmI to PlmIV but are not transported to the DV. Recently, it has been reported that PlmV licenses Pf proteins for export into the host erythrocyte, therefore, it is essential for parasite viability [41]. The remaining Plms (PlmVI, PlmVII, PlmVIII) are not expressed during the intraerythrocytic stage [38]. Plms from the other human-infecting parasites (*P. vivax*, *P. malariae*, and *P. ovale*) have also been identified and characterized (see [42, 43] for more detail). 

Although the degree of sequence identity among the aspartic proteases of *Plasmodium* species is relatively high, substrate specificity and their response to inhibitors differ, indicating that variations may exist in the protein-ligand binding interactions [40, 44–46]. Among *Plasmodium* species, only Pf strains possess genes encoding PlmI, PlmII and HAP. Furthermore, PlmIV has a higher level of sequence identity with plasmepsins from nonfalciparum species (65–76%) than their paralogues PlmI, PlmII, and HAP (63%, 62%, and 53%, resp.) [47]. In particular, PlmIV plays a crucial role, as it is the only Plm of Pf with orthologs in the other *Plasmodium* species that infect humans, and, therefore, opens a way to affect all the *Plasmodium* parasites with one inhibitor [47]. Considering PlmII as reference, PlmI shows 73% sequence identity, PlmIV 69%, and HAP 60%. These sequence identity values extend to the binding site region. In this case, PlmI shows 84% identity, PlmIV 68% identity and HAP 39% identity. HAP has the lower degree of identity despite most amino acid substitutions within the binding site are rather conservative (55% sequence similarity) [48]. The amino acid sequences of PlmI, PlmII, and PlmIV display the classic catalytic motif of aspartic proteases [49] present in one copy in the N-terminal and C-terminal domains [50]. Although the motifs are recognizable in the HAP sequence, they show unusual modifications the catalytic aspartate of the N-terminal domain is substituted by histidine, and both conserved glycines are replaced with alanines [51]. 

Structure-based drug design of antimalarial compounds targeting plasmepsin inhibition is possible due to the availability of several three-dimensional (3D) structures of these enzymes. Nineteen crystal structures of PlmII have been deposited so far, two of which correspond to the free enzyme (PDB: 1LF4, 3F9Q), one to the proplasmepsin (PDB: 1PFZ), and the others to protein-inhibitor complexes (PDB: 2R9B, 1W6H, 1W6I, 1LF3, 1LEE, 1EX5, 1EX6, 2BJU, 1ME6, 1LF2, 1SME, 1PFZ, 1XDH, 1ME6, 2IGX, 2IGY, 1M43). For PlmI, only one homology model has been described so far [18]. From *P. falciparum*, there are also deposited X-ray structures of PlmIV-inhibitor complex (PDB: 1LS5) and three of HAP; one structure of free HAP (PDB: 3FNS) and two complexes (PDB: 3FNT, 3FNU). In addition, structures from other species of *Plasmodium *have been reported: one of PlmIV from *P. malariae* (PDB: 2ANL) and two from *P. vivax* (PDB: 1QS8, 1MIQ). It should be noted that Plms form homodimers with extensive interfaces in most of the known X-ray structures; conversely, an experimental study revealed that PlmII exists mainly as a monomer in solution, and that the monomer is fully functional for catalysis [52]. Therefore, practically all the *in silico* studies of these enzymes use the monomer structure as target [53–58]; with the drawback, these proteins need an extensive computational work to relax the regions of the protein buried in the dimmer.

The redundant functional role of the Pf DV plasmepsins in Hb digestion has been demonstrated by knockout experiments [59–61]. This feature indicates that more effective drugs may be obtained by blocking more than one plasmepsin. However, recent experiments point out that plasmepsins are not essential for the parasite viability. Bonilla and colleagues demonstrated the slow growth of parasite mutants that lack all DV plasmepsins in amino-acid-limited medium [62]. On the other hand, Moura and coworkers showed that a wide range of previously characterized aspartic protease inhibitors exert their antimalarial activities primarily upon one or more non-DV plasmepsins and secondarily on the DV Plms [63]. 

## 3. Sequence and Structure Analyses of Plms Family

To quantify the structural variations in Plms upon ligand binding Bhargavi and coworkers, estimated the backbone global root mean square deviation (GRMSD) values between the residues of uncomplexed Plms and those bound to ligands, using crystal structures of PlmII and homology models for PlmI, PlmIV, and HAP [6]. These authors identified four loops that showed large structural deviations on ligand binding, which were denoted as L1, L2, L3, and L4, involving residues 12–14, 158–165, 231–244, and 277–283 (PlmII numbering scheme), respectively [6]. Moreover, the comparison of the recent HAP apoenzyme crystal and its pepstatin A complex with PlmII, PlmIV, human pepsin, and the complex human pepsin-pepstatin A, presented also pronounced differences in the conformation of the loops 238–245 and 276–283, corresponding to L3 and L4 regions [51]. Entropic analysis in Bhargavi's work from wormlike chain model for loops, along with the GRMSD values, indicated that L3 loop has an inherent tendency to lose entropy on binding in order to attain stability. However, in this paper, the authors proposed, based on the crystal conformations, that the residues of these regions have negligible electrostatics and nonpolar interactions with the inhibitors therefore, they highlighted the role of these loops in determining only the openness of the binding cavity.

**Figure 1:**
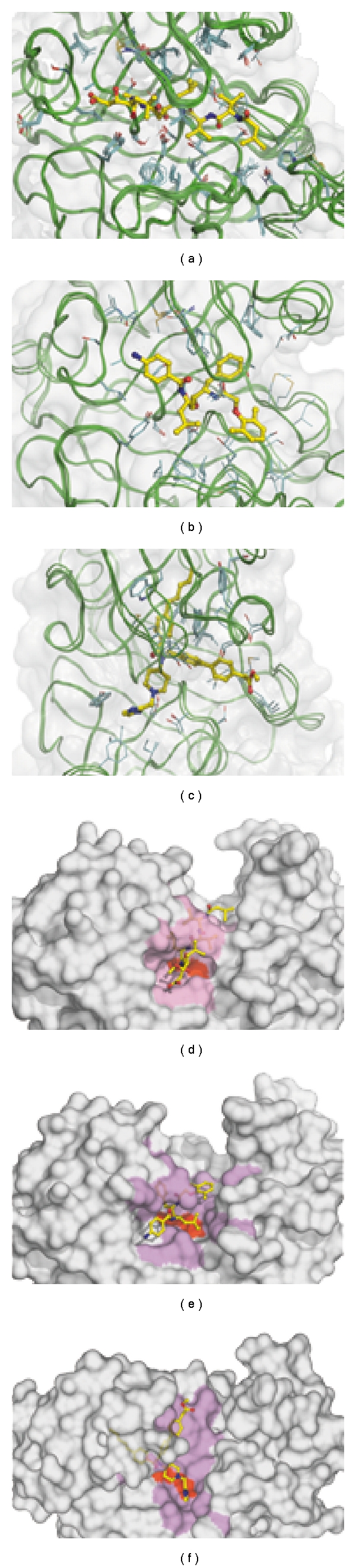
The different binding modes described for plasmepsin II*. Closed (a), partially opened (b), and open (c). Protein backbones are represented as green ribbons. Ligands (pepstatin A (a), rs370 (b), and IH4 (c) in yellow), and all amino acids in their close vicinity (up to 6 Angstrom) are shown in stick using a color code by the atom type. Panels D to F show the solvent accessible surface of a representative protein from each binding mode (PDB ID: 1XDH (a), 1LF2 (b), and 2BJU (c)). The zone up to 4 angstrom away from the ligands is colored in magenta, and catalytic residues D34 and D214 are shown in red. These figures were prepared with PYMOL. *According to Luksch and colleagues [4].

Recently, Luksch and colleagues classified 14 X-ray structures of PlmII complexes into three groups (Figure 1), exhibiting different ligand binding modes [64] (1) all complexes with pepstatin and pepstatin-like ligands, because as peptide mimetics, they exhibit an almost identical ligand binding mode (2) three complexes in which the binding pocket is in a partially open conformation and (3) three complexes with inhibitors featuring *n*-pentyl substituents, that address a new specificity pocket, the so-called “flap pocket.” The crystallographic indicated adaptivity of the protein is further confirmed by molecular dynamic (MD) simulations [4, 65–69]. Bursavich and Rich suggested that PlmII, and most likely PlmI and PlmIV, are highly flexible proteins that adopt additional conformations not yet characterized, but which could possibly be targeted by inhibitors [70]. Because only three distinct binding modes have been discovered so far, and due to the fact that the target protein has been treated as rigid in most *in silico* studies, the application of automated docking procedures appears rather limited. 

The feasibility of finding or designing an inhibitor capable of targeting several proteins with high affinity requires that the binding sites in all members of the target family share conserved regions against which the strongest interactions can be directed [45, 53]. Highly conserved residues within family or subfamily are strong candidates to be located in functional important sites. These residues are expected to be involved in determining the interaction specificity of subfamilies members in binding pockets and are generally referred as tree-determinant residues [71] or trace residues [72]. Ernesto Freire's group has probed the usefulness of these concepts in the design of *adaptive inhibitors *[53, 54]. Adaptive inhibitors establish their strongest interactions against conserved regions of the target, and contain flexible elements and asymmetrical functional groups that allow them to accommodate to variable regions within the target family [53]. Nezami and colleagues [53] used this principle to design an inhibitor with subnanomolar affinity (0.5 nM) primary against the PlmII, and with no loss or a very small loss of affinity against PlmIV, PlmI, and HAP (*K*_*i*_ ratios of 0.4, 7.1, and 17.7, resp.). To achieve this goal, the authors constructed a composite plasmepsin binding cavity by using the backbone of PlmII as a template and placing the side chains of the four plasmepsins at their corresponding site within the binding cavity. Some regions within the composite binding site included very conservative amino acid substitutions that altered only the shape but not the chemical polarity or charge of those regions; only a small area of the binding site contains substitutions with different polarities and none of them with opposite charge. In particular, the region corresponding to P1′ and P2, opposite the opening of the flap, showed significant variability. Another variable region found was the flap itself (residues 75–79). As expected, most of the variability was found in the HAP protease. These results concur with a previous work published by Nezami and Freire [54], who used entropic analysis to describe the variability at each position in a multiple alignment of Plms sequences. Once conserved and variable regions within the binding site have been identified, the next step in the design of an adaptive inhibitor is the identification of a molecular scaffold that establishes strong interactions with the most conserved regions of the target site. As the amino acid substitutions were found conservative and induced only a shape distortion in the binding site, adaptation was achieved by introducing asymmetric functional groups linked to the inhibitor core by rotatable bonds. Based on this information, Nezami and colleagues designed a series of allophenylnorstatine-based compounds, whose thermodynamic properties were experimentally tested with microcalorimetric analysis. Despite these efforts, most of these compounds having a poor selectivity respect the hCatD. 

**Figure 2:**
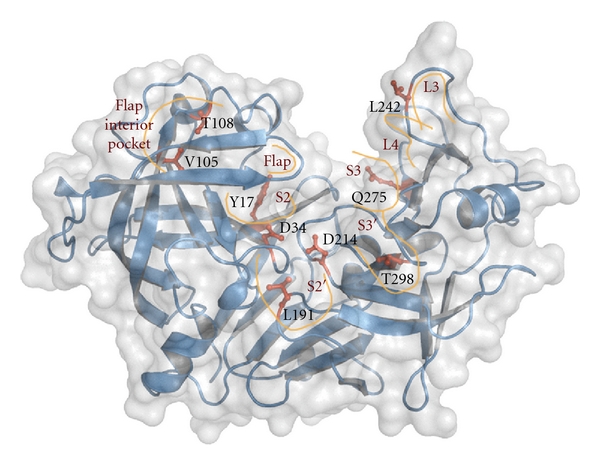
Top view of the surface representation of plasmepsin II. The seven new functional residues proposed by Valiente and colleagues [5], and the catalytic aspartic residues, are shown in stick representation. The enzyme subsites, the flap, and the flexible loops L3 and L4 (identified by Bhargavi and coworkers [6]) were sketched in orange.

In this respect, identifying the functional residues responsible for plasmepsin specificity could help the development of more potent and selective inhibitors. Recently, Valiente and colleagues [5] performed a multiple sequence alignment with 73 homologous amino acid sequences that show identity ranging from 10 to 88%, in order to define key residues for Plms activity. Based on this sequence analysis, combined with structural analysis, and MD simulations of Plms-ligand complexes, these authors predicted for the first time that residues Y17, V105, T108, L191, L242, Q275, and T298 (PlmII numbering scheme) could be important for the plasmepsins function (Figure 2). These 7 promising amino acid residues are conserved in the malarial strains but not among human aspartic proteases. Residues V105 and T108 are located in a loop of an interior pocket and only establish contacts with a specific nonpeptide achiral inhibitor, as was illustrated analyzing the PlmII-inhibitor X-ray structures. Residue L242 is located in the L3 loop, recently described as an essential region in cleaving intact hemoglobin [73]. Residue Q275 is situated in a small *β*-strand in close vicinity to the L4 loop. Finally, residues Y17, L191, and T298 belong to well-defined pockets lining the binding site cavity. In this work, the authors proposed a useful strategy that combines the information derived from the sequence and structural analyses with MD simulations of protein-inhibitor complexes, which gave good results applied to the prediction of residues with functional properties. 

Sequence-based methods [74] and visual inspection of active site are not sufficient for determining the selectivity of different targets. Limitations of those methods make understanding of contributions of various interactions in the binding process very difficult. The most popular method used for mapping selectivity is the molecular interaction fields (MIF) [75] implemented in the program GRID [76]. MIFs are calculated by placing chemical probes in the active sites of the protein. MIFs are produced in the form of interaction maps of the binding site, which indicate the most favorable regions for placing ligand groups with properties similar to the probes, thus generating complementary maps of the active site. Therefore, MIF is a collection of energy values calculated from the sum of the attractive and repulsive forces between a molecule (a target) and an interacting partner (the probe), positioned in a lattice of points (or nodes) surrounding the target. Nodes with negative energy values correspond to favorable interactions between the molecule and the probe. Kumar and Ghosh [77] characterized the binding site of four malarial aspartic proteases (PlmI, PlmII, PlmIV, and *P. vivax *plasmepsin), and two human aspartic proteases (hCatD and pepsin) with the intention of identifying the regions that could be potential sites for obtaining selectivity using a MIF approach. Their data showed that specificity was founded towards the region of amino terminal of the scissile bond of peptide substrate for example, in S1′-S1, S2, S3, and S4, while selectivity occurred towards the carboxyl terminal of that scissile bond, S1′-S1, S2′, and S3′. The pocket S3 was retrieved to be both selective and specific.

## 4. Popular Approaches Used in the Structure-Based Drug Design of Plms Inhibitors

The drug discovery process has changed during the last decades by the adoption of computational methods helping the design of new drug candidates more rapidly and at lower cost. *In silico* drug design consists of a collection of tools helping to make rational decisions at the different steps of the drug discovery process, such as the identification of a biomolecular target of therapeutic interest, the selection or the design of new leading compound, and their modification to obtain better affinities, as well as pharmacokinetic and pharmacodynamics properties. If spatial structure of target is known, the methods of structure-based drug design are applicable. Among the different tools available, a particular emphasis is placed in this review on the use of molecular docking, virtual high-throughput screening (HTS) and fragment-based drug design (FBD). 

Computer methods for drug design are based on a postulate that pharmacologically active compounds act by interaction with their macromolecule targets, mainly proteins or nucleic acids. To improve the knowledge about the target-ligand interactions and to predict the native position, orientation, and conformation of a small-molecule ligand within the binding site of a targeted macromolecule, several docking algorithms have been developed. Docking algorithms are combined with approximate methods for rapid estimation of the binding affinity, named scoring functions, needed to identify the “native” binding mode. Over 30 different docking programs are available today [27]. The most popular for docking, and currently used on Plms, include AutoDock [78, 79], Dock [80, 81], FlexX [82], FlexE [83], Glide [84, 85], and Gold [86, 87]. 

Although they exploit different strategies in the ligand placement, all of them can be categorized into four broad categories: stochastic Monte Carlo, fragment-based, evolutionary-based, and the shape complementary methods. A fragment based incremental method is represented by FlexX and Dock. In this approach, a ligand is split into fragments, which are docked independently, and then their molecule structure is recreated typically in an incremental way. The evolutionary methods are used in Gold and AutoDock. These two programs use genetic algorithms to perform the conformational search. Force field-based methods, like Glide, implement Monte Carlo-based engine. Finally, the complementarity shape methods, like LigandFit [88], exploit grids to fit the shape of a ligand into an active site of the target combined with Monte Carlo sampling. None of those programs use a systematical search to fully explore all degrees of freedom in the ligand molecule because of the enormous computational cost of such a procedure [89]. However, in order to take into account the conformational differences during the physical binding observed in the structural studies of receptor-ligand systems, it is necessary to include the intrinsic flexibility of the whole system. Therefore, the docking process is performed usually considering only a conformational space with a reduced number of degrees of freedom. For example, it is a common practice to apply some flexibility to the protein during the docking through active site side-chain rotations and more global minimization, or to use a set of different pre-generated receptor conformations obtained experimentally or with *in silico* approaches. At the end, the ligands are ranked relative to each other by a scoring function, a method that can estimate free energies of binding from structural information, or by purely energetic criteria, using a force field. Based on these evaluations, the compounds with the best complementarities to structure and properties are selected.

In a recent review [90], it was evaluated the perform of seven popular docking programs (Surflex [91], LigandFit [88], Glide, Gold, FlexX, eHiTS [92], and AutoDock), which enclose all the mentioned ligand pose methods, on the extensive dataset composed of 1300 protein-ligands complexes from PDBbind 2007 database, where experimentally measured binding affinity values were also available. The results obtained clearly showed that there was not single program that consistently outperformed all others. Nevertheless, programs that use genetic algorithms seem to be the best choice for the pose prediction; yet, due to the nature of the algorithm, docking takes much longer time than other types of algorithms. 

Drug development efforts targeting the plasmepsins have been facilitated by previous studies on other aspartic proteases, particularly renin and cathepsin, which have provided most of the inhibitors used in the crystallographic studies. There have been many works that applied to the plasmepsins case some of the above-mentioned docking software; among the most used are AutoDock [29, 93–96], Gold [55–58], and FlexX [29, 64, 67]. These programs have been employed principally to obtain complexes structures that helped to the interpretation of the experimental results; for the screening of different combinatorial libraries of ligands and to generate plasmepsin-substrate conformations used to establish the enzyme reaction mechanism [97]. Also, they allow the rationalization of the inhibitors potency in terms of structural parameters (like number of H-bonds or contact surface area). Moreover, there are some reported cases in which rigid docking failed to predict reasonable binding modes based on previously determined crystal structures [66, 68], therefore, a manually docking approach was used instead. This problem was adjudged to steric reasons if the binding pocket in the X-ray structures was partially collapsed [66], or when it was used as a homology model of starting protein structure [98]. Consequently, some researchers have used a combination of manual inhibitor adjusting and docking, or just a hand-generated structure refined with a molecular mechanics minimization [4, 53, 66, 68, 99]. For example, Beyer and colleagues [94] investigated the possible binding modes of a group of designed peptidomimetic inhibitors, using the crystallographic coordinates of the inhibitor rs370 as a reference, to manually adjust and approximate the position of their ligands, assuming that the hydrogen bond network was conserved for the backbone of the ligands. Those starting structures were subsequently refined trough a set of restricted docking calculations with AutoDock. The computational analysis employed in that work was able to tell the overall trend in apparent inhibition and to show that good experimental inhibitors interact with the plasmepsin active site through a mixture of hydrophobic and polar interactions. The manually docking was employed by other researches with good results, and of course, less consuming computing time. In general, the manual docking approach used previously consisted in superimposing the entire inhibitor backbone onto that of a similar ligand using a previously crystallized structure. The side chains of the ligands were then fitted to each corresponding subsite to minimize steric clashes. 

The scoring functions typically implemented in protein-ligand docking can be divided into three major categories [27]: knowledge-based (e.g., ITScore [100], PMF [101], DrugScore [102]), empirical (e.g., FlexX, Glide, Ludi [103, 104], ChemScore [105], X-Score [106]), and force field-based scoring functions (e.g., Dock, AutoDock, Gold, SYBYL/G-Score [86]). The first two methods suffer from a limited description of the physical aspects of the binding process and from a dependence on the experimental dataset used for their parameterization. On the other hand, the force field-based methods are universal, usually it is employed in a continuum solvent model to include the desolvation-free energy contribution (e.g., LIECE [107], Dock(PB/SA) [108]), but it does not take into account entropic effects. Although they are easy to use and can screen large libraries of compounds, they have difficulties in ranking ligands with small differences in chemical structures, for example, in lead optimization. The resulting binding affinities from scoring functions are often associated with errors of the order of 2.5 kcal/mol [109]. Some scoring functions that have been applied to plasmepsins inhibitor binding affinity prediction and ranking are X-score used in the characterization of the PlmIV binding site [57], Chemscore [105, 110]—GoldScore [86] both utilized in parallel docking runs [58, 111, 112], and the score functions of FlexX. In this respect, Ersmark and coworkers applied the Chemscore to complexes of PlmII and *C*_2_ symmetric peptidomimetic inhibitors [66]. They tested the accuracy of the functions against a group of allyloxy and benzyloxy stereoisomers, using single manual-docking minimized structures and MD ensemble averages. The scoring of single minimized complexes between the enzyme and the inhibitor resulted in energies that score the allyloxy stereoisomers incorrectly, and on the contrary it performed satisfactorily on the benzyloxy compounds. By averaging the score over 100 snapshots, the scoring function managed to rank the isomer series according to the binding affinity. The improvement of a scoring function results by taking the average over a structure ensemble, it had been shown previously [113]. Although the average results correctly rank the affinity within the isomer series of inhibitor analyzed, it largely overpredicts the affinities of a group of ligands due to the lipophilic term of the scoring function, which clearly overestimates the hydrophobic binding contribution (predicted to about −10 kcal/mol). The authors indicated that this could represent a more general problem with scoring functions that had a built-in size dependency of the hydrophobic term through surface area or similar size measures. This procedure was successfully applied also to PlmIV with a group of isosteres using X-score. The binding estimates were also very good in the relative ranking of the compounds. The absolute values for the predicted affinities, however, were shifted by an average overprediction from 2.7 to 3.0 kcal/mol depending the PlmIV structure employed. It must be appreciated the application of X-score, in that work, to the analysis of the inhibitors selectivity, measured as the differential binding free energy (ΔΔ*G*_bind_) between PlmIV and PlmII. The strong selectivity of the most potent inhibitor for PlmIV was not particularly well reproduced. Ludi is another scoring function applied to PlmII and PlmIV using HIV-1 inhibitors, which gave a reasonable agreement with experimentally inhibitor potencies [112]. Nevertheless, it ranked poorly the X-ray complex of PlmIV-pepstatin A (PDB:1LS5), which was not adjudged to an optimal filling of the inhibitor sidechains on the protein subsites. One of the advantages of the application of empirical scoring functions is its simple relationship between structural parameters, like hydrogen bonds and lipophilic interaction energy, with enzyme inhibitory potency of the compounds. This issue lead to a valuable information for inhibitor design, but it must be taken carefully, since these relations are, in the best cases, of a qualitative value. 

Despite a good number of scoring functions that have been developed, none of them is perfect in terms of accuracy and general applicability. To take the advantages and balance the deficiencies of different scoring functions, the consensus scoring technique has been introduced to improve the probability of finding correct solutions by combining the scores from multiple scoring functions. The critical step in consensus scoring is the design of an appropriate combining strategy of individual scores so that the true modes/binders can be discriminated from others accordingly [109]. As alternative to the scoring function, a new combined docking workflows—AutoxX—unifies the interaction models of AutoDock and FlexX rather than combining the scores afterward which allows interpretability of the results [114]. The application of this strategy to 4 plasmepsin experimental complexes achieved an improvement of the root mean square deviation (rmsd) of predicted conformations versus the corresponding native one over AutoDock and FlexX of 1.70 against 3.98 and 7.09, respectively. 

**Figure 3:**
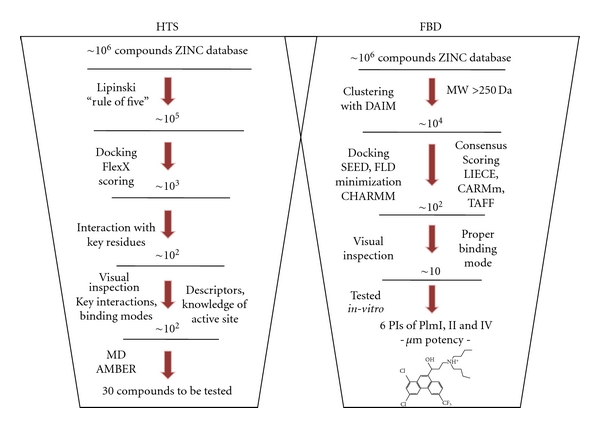
Overall scheme of *in silico* screening strategy applied to Pf plasmepsins. FBD represents the fragment-based docking process performed by Friedman and Caflisch [28], and HTS the high throughput screening carry out by Kasam and colleagues [29]. Details can be revised in the corresponding references.

A high-throughput screening is typically used at an early stage of the drug design process in order to test a large compound collection for potential activity against the chosen target [115–117]. Unfortunately, this method is time-consuming and expensive. For this reason, virtual HTS has become an important tool to precede the large *in vitro* screening assays [118–120]. This method aims at using computational tools to estimate *a priori*, from an entire database of existing (or hypothetical) compounds, those that are the most likely to have some affinity for the target. The general techniques are random screening and generation of focused libraries based on pharmacophore with subsequent screening of the resulting virtual compound. A current application of this methodology to design of new plasmepsin inhibitor was reported by Kasam and coworkers, in the frame of the WISDOM (Wide In Silico Docking On Malaria) project [29] (Figure 3). They developed a computational random screening approach, based on a high-throughput molecular docking and implemented it at large scale on the EGEE grid infrastructure [28]. In the mentioned study, structures of PlmII and PlmIV were used with or without crystal water molecules, to check the influence of waters in the docking scores. As a result, for the protein structure 1LEE with crystal water molecules, all ligands failed to form interactions with the key residues. A total of 1,000,000 compounds were downloaded from ZINC database and docked into plasmepsins using FlexX. One crucial step of the virtual HTS is the result analysis, due to the difficulty of scoring functions, like the usually emergence of top scoring compounds with poor binding modes or which failed to form the expected interactions to key residues of the protein. After undergoing a filtering procedure to the docking solutions, 100 compounds were selected by Kasam and coworkers for reranking by molecular simulations. Most of the compounds selected were thiourea, guanidino analogues, and diphenyl urea; the last known micromolar inhibitors of plasmepsin [93]. By utilizing the same procedure, docking software and chemical compounds from the ZINC database, the same researches performed large-scale virtual screening against four different proteins implicated in malaria producing short lists of particularly promising molecules [121]. If human proteases are including the screening, this kind of computational initiative could give an important contribution to the development of potent and selective inhibitors.

Since a few years, FBD has become an attractive alternative to the experimental or virtual HTS [122]. Contrary to virtual HTS, where complete molecules are screened for activity, FBD aims at building new ligands piece by piece by connecting small and well-chosen compounds that bind into separate binding pockets, close enough to be chemically linked in their relative favorable positions [123]. Haque and colleagues [36] used a combination of FBD and synthetic combinatorial library design to find potent and low-nanomolar inhibitors of PlmII. The “anchor and grow” algorithm implemented in Dock was employed to model each scaffold and side chain from a virtual library in the PlmII active site and scores consisted of van der Waals and electrostatic terms from the AMBER force field [124]. Recently, Friedman and Caflisch used FBD procedure to search for inhibitors of PlmII [125] (Figure 3). A total of 4.6 million compounds were first clustered according to 2D structural similarity resulting in about 40,000 molecules which were then used for FBD. Docking into the PlmII active site was followed by consensus scoring using four force field-based energy functions. A total of 19 compounds were tested in an enzymatic assay, and three of them showed single-digit micromolar inhibitory activity. One of these three inhibitors was halofantrine, an antimalarial drug discovered more than 40 years ago whose mechanism of action is still unknown. To better investigate the binding mode of halofantrine, four 50 ns MD simulations with explicit solvent were performed starting from two different poses, one generated by automatic docking and the other by manual fitting with the help of a computer graphics program. The MD simulations indicated that the binding mode generated by fragment-based docking was more stable than the one obtained by manual docking, although it was not possible to definitively discard either one.

## 5. Calculation of Absolute Binding Free of PlmII-Inhibitor Complexes Using the LIE Method

**Figure 4:**
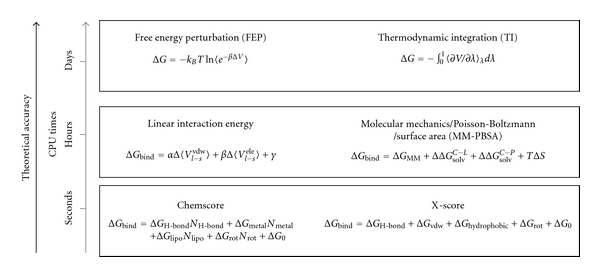
Theoretical approaches used to calculate the binding free energy of protein-inhibitor complexes. The arrows show the correlation between theoretical accuracy of methods and the computational time required to perform the calculation.

One of the main challenges in computational structure-based ligand design is the estimation of absolute binding affinities for ligand-receptor complexes. Several approaches to this problem have been developed (Figure 4), ranging from empirical and “knowledge-based” scoring functions to those based on free energy calculations, such as the rigorous free energy perturbation (FEP) and thermodynamic integration (TI) methods [126, 127]. However, FEP and TI approaches are quite time consuming, and the associated sampling and convergence problems limit their use to relative binding free energy calculations between pairs of molecules having only minor structural differences [126–128]. Thus, further development of fast and accurate methods for structure-based drug design is still needed. Åqvist and coworkers developed a semiempirical method [30] termed as the linear interaction energy (LIE) approximation, for the estimation of absolute binding free energies. This method, which is based on conformational sampling by MD or Monte Carlo (MC) simulations, is faster than FEP and TI, since it avoids sampling of any unphysical intermediate states between the initial and final configurations. However, it is considerably slower than single-conformation scoring function methods [30]. LIE has been successfully applied in several projects addressing ligand binding as well as protein-protein interactions [126, 129, 130], and has inspired other related methods [107, 131–133]. Two examples are SGB-LIE [133] and LIECE, both of which treat the solvent as a continuum, compared to LIE where water molecules are explicitly represented. The LIE method is based on the linear response (LR) assumption for electrostatic interactions with an empirical expression for nonpolar effects. In this approach, the binding free energy is estimated according to (1):

(1) $$\Delta G_{\text{bind}}=\;\alpha \Delta \langle V_{l-s}^{\text{vdw}}\rangle +\;\beta \Delta \langle V_{l-s}^{\text{ele}}\rangle +\gamma ,$$

where 〈*V*_*l*−*s*_^vdw^〉  and 〈*V*_*l*−*s*_^ele^〉  denotes MD or MC averages from the nonbonded van der Waals and electrostatic interactions of the ligand (*l*) with its surrounding environment (*s*), respectively. The Δ's denote the change in average values when transferring the ligand from solution (free state) into the binding site of the solvated receptor (bound state). The coefficients *α* and *β* are scaling factors for these energy terms, while *γ* is a constant correction term sometimes proposed to represent entropic contributions to the free energy of binding of different types of receptor sites [134, 135]. The LR approximation theory provides a physical basis for the treatment of the electrostatic contribution to the binding free energy, which predicts a value of *β* = 0.5 [30, 136]. The assumption that nonpolar ligand-surrounding van der Waals energies (represented by a Lennard-Jones potential) can be used to calculate the nonpolar contribution to binding free energy is based upon the observation that the salvation-free energies of nonpolar compounds scale linearly with molecular size descriptors such as surface area [30]. However, it is not a straightforward task to predict the *α* or *γ* values from theoretical considerations, being usefully obtained as empirical parameters by fitting to experimental data on small set of receptor-ligand complexes [129, 130, 134, 135, 137, 138].

The LIE method usually employs MD simulation averaging of the intermolecular interactions between the ligand and its surrounding environment in the two relevant states, for example, the ligand solvated in water (free state) and the solvated protein-ligand complex (bound state). MD sampling of the protein-ligand complex allows structural and energetic relaxation of the starting structures. This is a major difference compared to the use of scoring functions, where binding energy is usually determined from a single energy minimized receptor/ligand complex. Prior to all simulations, the ligand or the ligand-protein complex was solvated with explicit water molecules, and restrained spherical simulation boundaries were used in all calculations [19, 26, 55, 56].

Like other semiempirical methods, the success of LIE resides principally in the selection of the parameter values. Several parameter schemes have been developed so far [139, 140]. The empirical coefficient *α* was initially calibrated against the experimental binding data on a small training set of four endothiapepsin inhibitors of similar scaffold using *β* = 0.5 [30] giving *α* = 0.161 with a version of the Gromos96 force field [141]. This original model yielded reasonable binding free energy estimates for different proteins in complexes with ligand of dissimilar scaffolds such as endothiapepsin [30] HIV-1 protease [142, 143], glucose binding protein [144], and trypsin [145]. This parameterization was subsequently refined by Åqvist and coworkers [134, 135] using results from simulations of 18 protein-ligand complexes of the same proteins as training set. These authors determined the specific *β* values using FEP calculations, motivated by systematic deviations from the linear response theory observed for dipolar group compounds [134, 146]. As a result, it was obtained an improved LIE model, which included *β*_FEP_ values ranging between 0.33 and 0.5, along with *α* = 0.18 and *γ* = 0, that resulted in calculated binding free energies in good agreement with experimental data for several protein-ligand systems [58, 65, 66, 147, 148]. However, in other cases, a nonzero *γ* constant term is required to reproduce the experimental absolute binding free energies [130, 137, 138]. Some notable cases are the binding of retinoids to retinol binding protein (RBP) [130], biotin analogs to avidin [137], substrates to cytochrome P450 (P450cam) [138], and inhibitors to human thrombin [149]. For these systems, the *γ* values ranged from −2.9 kcal/mol to −7 kcal/mol [129]. Recently, Almlöf and colleagues [147] found a clear relationship between the ordering of hydrophobicity ranking of these binding sites (RBP > P450cam > thrombin > trypsin) and the value of *γ*. To some extent, this is similar to the idea developed by Wang and coworkers [137], who investigated variations of the nonpolar coefficient *α* in the absence of the constant term *γ*, as a way to distinguish between different types of binding sites. The main outcome is the linear correlation obtained between the weighted nonpolar desolvation ratio (WNDR) and the values of *α* in the LIE method. Briefly, the WNDR was defined [137] as the ratio of all nonpolar groups' weighted desolvation solvent accessible surface area (SAS), carbon and sulfur atoms in this case, to the total weighted desolvation SAS. The WNDR parameter can be useful to predict the value of *α* for those systems in which very different ligands bind to the same protein, as a way to distinguish between different ligand binding modes or when these ligands bind to different sites of the same protein [137].

The standard parameterization of LIE (*β* = *β*_FEP_, *α* = 0.181, and *γ* = 0) has been applied with excellent results to predict the binding free energies of Plms in complexes with inhibitors based on the 1,2-dihydroxyethylene scaffold [58, 65, 66, 68, 111]. Conversely, in recent LIE studies were not able to reproduce the absolute binding affinities of PlmIV in complex with inhibitors based on the *α*-phenylnorstatine [55, 150] and *α*-benzylnorstatine [55] scaffolds, and macrocyclic inhibitors (PlmII and PlmIV). In addition, Valiente and colleagues [140] reported that the standard parameterization of LIE failed to reproduce the experimental binding free energy of PlmII in complex with achiral (IH4 [151]) and hydroxyethylamine/hydroxypropylamine (EH58 [152]/rs367 [153], rs370 [153]) inhibitors. However, in this study, the absolute value of the binding free energy of PlmII in a complex with pepstatin A was in agreement with the experimental data. This fact suggests that the possible dependency of PlmII-inhibitor system on the LIE method might be circumvented by using higher values of the nonpolar scaling factor *α*, or alternatively, by the addition of different nonzero *γ* constant term for each PlmII-ligand binding mode [140]. To achieve this goal, these authors developed three different approaches of the LIE method, to predict binding free energies by combining different approaches to estimate *α*, *β*, and *γ* parameters. The best model combined an optimized *α* parameter, calculated from the second one parameterization model of WNDR versus *α*, while setting *γ* = 0, and *β* according to model E proposed by Almlöf and colleagues [139]. Their results agreed well with the experimental data and the chemical nature of the inhibitors assessed. In addition, Valiente and colleagues also showed that the WNDR parameter yielded better results for proteins with high structural flexibility, when it was applied to the fitting of the *α* parameter rather than *γ*. The analysis of calculated interaction energies for these training set showed that the nonpolar contribution (Δ*G* ^*np*^ = *α*〈*V*_*l*−*s*_^vdw^〉) of each PlmII-inhibitor complex was always favorable to binding. An earlier study showed that nonpolar interactions gave the largest contributions to absolute binding affinities of inhibitors based on the 1,2-dihydroxyethylene scaffold [66]. Although the van der Waals interaction energies from the complexes used by Valiente and colleagues looked quite similar, the nonpolar contribution to the binding affinities showed striking differences among pepstatin A and the other inhibitors (rs367, rs370, EH58, and IH4). Since the *α* scaling factor took into account the fraction of the enzyme interacting with the inhibitors, the lower percentage of enzyme nonpolar groups desolvated after pepstatin A binding determined the less favorable nonpolar contribution from this inhibitor to protein association. In contrast, the electrostatic interaction energies from these inhibitors showed that pepstatin A has only a favorable electrostatic contribution to binding. 

## 6. Concluding Remarks

The structure-based drug design of antimalarial compounds targeting *P. falciparum* DV plasmepsin inhibition has received much attention in the last 15 years due to their potential biomedical use. However, a recent study showed that a wide range of previously characterized aspartic protease inhibitors exert their antimalarial activities primarily on one or more non-DV plasmepsins and secondarily on the DV plasmepsins [63]. This finding indicates the relevance in the intraerythrocytic stage of the non-DV plasmepsin as PlmV [41], PlmIX, and PlmX, although these enzymes have not 3D structures solved by experimental methods yet.

Despite some limitations, the combination of docking algorithms with the LIE method constitutes a good starting point to develop new potent and selective plasmepsin inhibitors. In this respect, identifying the functional residues responsible for plasmepsin specificity could help to achieve these goals. Moreover, we consider that new aims as the identification of novel conformational states of Plms (non-DV and DV) that cannot be adopted by the human aspartic proteases need to be addressed and would be useful for inhibitors design.

##  Conflict of Interests

The authors declare no conflict of interests.
